# Using the Fingerprinting Method to Customize RTLS Based on the AoA Ranging Technique

**DOI:** 10.3390/s16060876

**Published:** 2016-06-14

**Authors:** Bartosz Jachimczyk, Damian Dziak, Wlodek J. Kulesza

**Affiliations:** 1 Faculty of Electrical and Control Engineering, Gdansk University of Technology, G. Narutowicza 11/12, 80-233 Gdansk, Poland; damian.dziak@pg.gda.pl; 2 Department of Applied Signal Processing, Blekinge Institute of Technology, 371 79 Karlskrona, Sweden; wlodek.kulesza@bth.se

**Keywords:** accuracy and precision, angle of arrival, calibration point, fingerprinting method, indoor localization systems, uncertainty map, real-time locating systems

## Abstract

Real-time Locating Systems (RTLSs) have the ability to precisely locate the position of things and people in real time. They are needed for security and emergency applications, but also for healthcare and home care appliances. The research aims for developing an analytical method to customize RTLSs, in order to improve localization performance in terms of precision. The proposed method is based on Angle of Arrival (AoA), a ranging technique and fingerprinting method along with an analytically defined uncertainty of AoA, and a localization uncertainty map. The presented solution includes three main concerns: geometry of indoor space, RTLS arrangement, and a statistical approach to localization precision of a pair of location sensors using an AoA signal. An evaluation of the implementation of the customized RTLS validates the analytical model of the fingerprinting map. The results of simulations and physical experiments verify the proposed method. The research confirms that the analytically established fingerprint map is the valid representation of RTLS’ performance in terms of precision. Furthermore, the research demonstrates an impact of workspace geometry and workspace layout onto the RTLS’ performance. Moreover, the studies show how the size and shape of a workspace and the placement of the calibration point affect the fingerprint map. Withal, the performance investigation defines the most effective arrangement of location sensors and its influence on localization precision.

## 1. Introduction

Emerging wireless technologies increase the potential and effectiveness of wireless Indoor Localization Systems (ILSs). Hence, recently, Real-time Locating Systems (RTLSs), an example of ILS, have gained increasing attention, mostly in the industrial sector, due to their capabilities and performance. RTLSs have the ability to locate the position of an item anywhere in a defined space at a point in time that is, or is close to, real time [1]. As fast-acting ILSs enhance safety, they become highly important in security and emergency systems. Moreover, they are widely used in healthcare facilities [2,3], social, and home care applications [4] for precisely tracking the movement of people. Besides that, RTLSs are widely applied in assets by tracking management systems in warehouses [5], container terminals [6], and hospitals [3].

Due to the radio wave propagation phenomena caused by environment structures and movements in indoor spaces, many wireless technologies based on Received Signal Strength (RSS) have, in practice, unreliable measurement methods. RSS-based ILSs are also disqualified from security applications, where attackers can influence the signal strength by attenuating or amplifying the signal. The usefulness of time-measurement-based localization systems is also limited because of hardware requirements. The Time of Arrival (ToA) method requires a highly accurate system clock and a precise synchronization module, which increase the system’s cost significantly. Therefore, Angle of Arrival (AoA)-based RTLSs seem to be a reliable and cost-friendly solution.

The fingerprinting technique is a pattern-based indoor localization technique useful for characterizing an indoor environment. It is established on matching measurement data with a previously defined pattern for instant and uncertainty pattern characterizing of a specific indoor environment. However, such patterns may take various forms, depending on the pattern nature. In the case of furnished indoor spaces, the pattern concerns structures and the arrangement of objects in the space. For people monitoring, it may include movement and an attendance probability map.

This paper proposes an analytical method for enhancing the performance of indoor RTLSs. The AoA ranging technique and fingerprinting technique, along with an analytical uncertainty analysis, are applied.

An implementation of the proposed customized RTLS method validates the analytical approach to fingerprinting mapping. The presented results of simulation and physical experiments verify the proposed method.

## 2. Survey of Related Works

RTLSs are usually in the form of radio frequency communication systems. They are able to localize a target in indoor spaces with relatively high accuracy, up to 15 cm for typical Ultra Wideband (UWB)-based ILS. An RTLS’s performance highly depends on the system architecture and configuration, as well as on the localization algorithm and method. Depending on the application and indoor environment character, ILSs may operate based on many different algorithms and the most common are proximity trilateration, angulation, fingerprinting, and dead reckoning. Localization methods producing ranging calculations are related to characteristics of measuring signals, such as RSS, ToA, and AoA [7].

The AoA ranging technique, a versatile method of the RTLS widely used in indoor environment applications, applies direction-sensitive antennae as a location sensors to estimate the direction (the angle) of the signal from a tag [8]. Kim *et al.* propose an improvement of the AoA-based RTLS on the application of a Dual Indirect Kalman Filter and weight filters, enhancing the estimation of the target’s position [9]. The hybrid algorithm is designed for Non-line-of-sight (NLoS) environments. In [10], the localization algorithm utilizes a biased estimation technique to increase the system performance. Moreover, the authors apply a statistical calibration method to improve the localization quality.

AoA-based localization techniques are also applicable in Wireless Sensor Networks (WSNs). In [11], the authors combine a hybrid AoA with the ToA method for localization purposes in a mobile WSN. The algorithm contains a particle-filtering module combined with an adaptive fuzzy controller. The multi-hop localization method is proposed by Park *et al.*, who base the AoA ranging method on iterative calculations of mutual distances and relative angles between neighbor nodes [12]. To increase performance of AoA-based WSNs, Y. Wang and K.C. Ho propose an AoA source localization estimator, which handles the sensor position error in WSNs [13].

Localization uncertainty is the crucial parameter of an RTLS’ performance and the key factor limiting the performance in indoor environments. Measurement accuracy of AoA antennas of RTLSs affects the quality of the localization estimation. Therefore, the Dilution of Precision (DoP), the concept originally used in satellite navigation systems, is derived for AoA-based positioning systems to characterize the positioning quality [14]. In [15], the authors show an impact of signal interferences on location accuracy in multipath environments. Cho *et al.* [6] apply an enhanced trajectory estimation method to estimate the track of mobile equipment with reduced uncertainty applied in container port terminals. The algorithm consists of “interpolation mechanism used to overcome omitting location estimation” caused by packet loss and an enhanced Kalman filter to correct the estimation error caused by the multi-path effect. Since the RTLS’ performance depends on a type and characteristic of antenna, and also on a type of indoor environment, then the antenna, properly matched to the indoor environment, may significantly improve localization accuracy [16]. The localization accuracy is also increased by optimal placement of the calibration emitter, which was investigated in [17].

To characterize an indoor environment, one can apply a suitable environmental pattern. Spatial AoA patterns may depict interferences caused by environment and localization factors and indicate sensitive areas. In [18], the evolutionary algorithm with the AoA ranging technique is used for selecting sampling points in multi-obstacle environments, and for this purpose the indoor pattern is established. The evolutionary algorithm is used to choose optimal Line-of-sight (LoS) measurement positions. A spatial pattern in the form of a channel impulse response map, which characterizes an indoor environment, is proposed in [19]. The pattern using a single-access point is applied as a database in the real position estimation process.

Fingerprinting is another pattern-based indoor localization technique useful for characterizing an indoor environment. Fingerprinting is based on matching measurement data with a beforehand-established pattern; moreover, a classification algorithm needs to be implemented. In [20], the authors present the WiFi-based RTLS using the fingerprinting algorithm with the 𝐾-nearest neighbor method to estimate the unknown location. Also, Artificial Neural Networks are widely applied in fingerprinting ILSs due to their pattern-matching features [21]. To enhance the fingerprinting-based location system in terms of localization accuracy, Kalman filtering has also been proposed [22].

## 3. Objectives and Main Contribution

In radio wave-based ILSs, interferences in signal propagation influence localization quality, which affects the system performance. Indoor environment specifics are important causes of the interferences. The geometry and structure of an indoor space, furnishing, and other obstacles influence the localization process. Customization of the RTLS’ algorithm concerning the indoor environment may reduce the impact of these interferences and then reduce localization uncertainty. From the review of related works, one can see that customization of the RTLS’ structure can be performed by creating an indoor environment pattern consisting of uncertainty map and sensors deployment. However, an analytical approach to establishing an uncertainty map that would correspond to the specified indoor environmental pattern is lacking.

The main objective of the paper is to develop an analytical method to customize RTLSs by improving the performance of indoor RTLSs based on the AoA ranging technique. The proposed method applies a fingerprinting technique with the defined uncertainty pattern in the form of an AoA localization uncertainty map. To establish such a map, an analytical method estimating the uncertainty of the AoA is needed. To address this task, a localization-uncertainty estimation method has to be established. The proposed estimation method uses a statistical approach to the AoA precision of a pair of specified location sensors, geometry of indoor space, and RTLS arrangement.

The main contribution of the paper is modelling of the fingerprinting technique. The model consists of ranging techniques and the uncertainty pattern of the AoA. To verify the RTLS model it is implemented in Matlab on indoor test environments. The performance of the models is evaluated and validated by results from physical experiments.

## 4. Customized RTLS Method Using AoA

**Figure 1:**
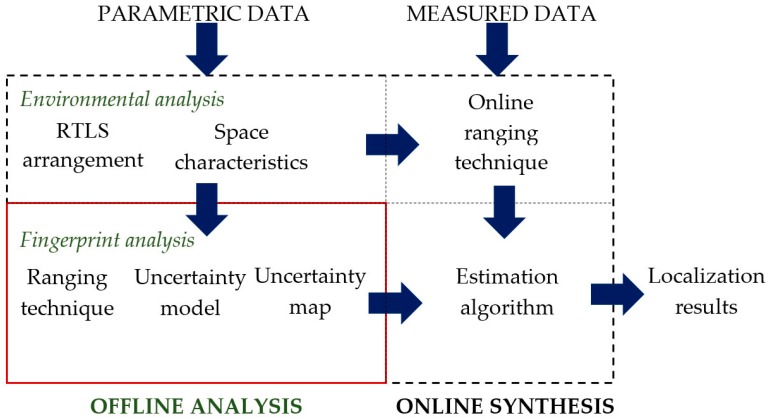
RTLS (Real-time Locating System) AoA (Angle of Arrival) fingerprinting method block diagram.

The proposed customized RTLS method applies the fingerprinting technique based on the comparison of the measurements with the established pattern. The method is illustrated in block diagram in Figure 1.

First, the offline analytical phase of customization consists of environmental and fingerprint analyses. The environmental analysis applies parametric data, such as shape and size of workspace, deployment of sensors, and location of the calibration tag, *etc.* The analytical phase is used to define environmental characteristics needed in the further steps. The workspace model is employed for the fingerprint analysis, where an uncertainty map is derived from the ranging technique model and the localization uncertainty distribution model.

Then, the environmental model and uncertainty map are implemented into the online synthesis phase when measured AoA data are processed. The synthesis applies the AoA ranging technique, estimating the relative tag’s positions in relation to all pairs of location sensors. The following estimation algorithm is used to find out the final tag’s position from a set of considered locations and the AoA uncertainty map defined in the fingerprint analysis stage. The method is described in the following sections.

### 4.1. Ranging Technique Model

The AoA ranging technique, simplified to a 2D azimuth approach, is implemented in both phases of the proposed customization-based method, in an offline analysis to establish the uncertainty map, and in an online synthesis phase to process actual measured AoAs.

**Figure 2:**
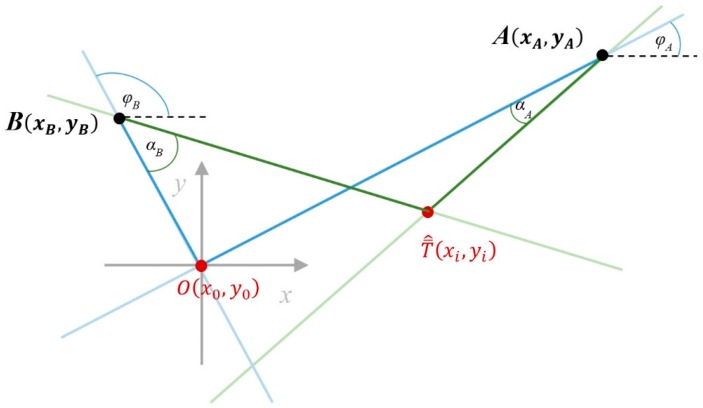
Graphical interpretation of the AoA ranging technique.

The AoA ranging technique is used to establish the tag’s position by estimating distances from the tag’s position to each LS (Localization System) placed in its fixed position. This technique is based on measurements carried out by LSs equipped with antenna array elements able to measure the AoA of radio waves emitted by the tag. Its geometrical interpretation is shown in Figure 2. In our approach, AoAs are angles (in Figure 2, angles $\alpha_{A}\;$and $\alpha_{B}$) between the line connecting a tag with a given LS, and the calibration line passing through the LS and the calibration point. The calibration point, O, is the coordinate system origin established in a calibration procedure. At least two AoA measurements are required to establish the tag’s position in a 2D scenario. 

On the basis of the installation data, during the calibration procedure, the workspace geometric shape is defined and the related positions’ coordinates, $A\left(x_{A},y_{A}\right)$ and $B\left(x_{B},y_{B}\right)$, of the LSs are established. Moreover, the calibration procedure defines the calibration point’s O coordinates $\left(x_{O},y_{O}\right)$ as the origin of the coordinate system. Furthermore, calibration angles $\varphi_{A},\;\varphi_{B}$, between the abscissa or horizontal axis and the calibration lines are depicted as:

(1) $$\varphi_{A}=\tan^{-1}\frac{y_{A}-y_{0}}{x_{A}-x_{0}}$$

(2) $$\varphi_{B}=\tan^{-1}\frac{y_{B}-y_{0}}{x_{B}-x_{0}}$$

Since the coordinates of the LSs of each pair are known, each LS provides measures of AoAs $\alpha_{A},\;\alpha_{B}$, which are referred to as primary established calibration lines. The coordinates $\left(x_{i},y_{i}\right)$ of the tag position $\hat{\bar{T}}$ can be estimated as an intersection of two lines determined by angles of arrival $\alpha_{A},\;\alpha_{B}$ to the respective LS [8]. Therefore, the tag position $\hat{\bar{T}}\left(\hat{x_{i}},\hat{y_{i}}\right)\;$can be calculated using the following formula [14]:

(3) $$\left[\begin{array}{l} x_{i}\\ y_{i} \end{array}\right]={\left[\begin{array}{ll} \tan\left(\alpha_{A}+\varphi_{A}\right) & -1\\ \tan\left(\alpha_{B}+\varphi_{B}\right) & -1 \end{array}\right]}^{-1}\left[\begin{array}{l} x_{A}\cdot \tan\left(\alpha_{A}+\varphi_{A}\right)-y_{A}\\ x_{B}\cdot \tan\left(\alpha_{B}+\varphi_{B}\right)-y_{B} \end{array}\right]$$

### 4.2. Offline Analysis

The offline analytical stage covers two steps: an environmental analysis and a fingerprint analysis. To define the uncertainty map, the indoor environment characteristics, ranging technique, and uncertainty distribution models are applied.

#### 4.2.1. Modelling of Indoor Environment

Performance of an indoor RTLS depends non-exclusively on the system arrangement and the space characteristics. The geometry of space and sensor arrangement affect the performance of the wireless indoor localization, due to interferences and reflections phenomena. 

**Figure 3:**
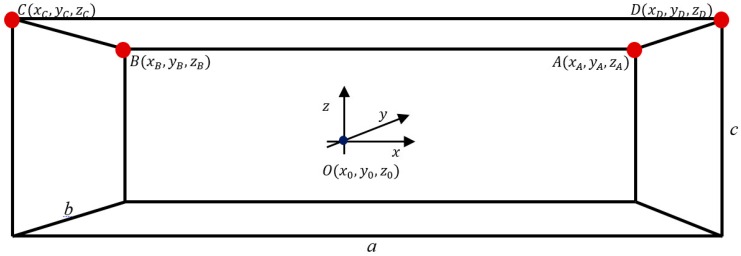
Model of 3D indoor environment.

The modeled indoor environment as an RTLS workspace presented in Figure 3 is a 3D rectangular cuboidal indoor space with an aspect ratio of its longer side *a* to its shorter side *b* and height *c.* The coordinate system’s origin $O\left(x_{0},y_{0},z_{0}\right)$ corresponds to the calibration point, where the calibration tag was located. The calibration tag needs to be located in the LoS of all LSs, however, the most desirable location is the central area of the workspace.

The optimal deployment of LSs in a 3D indoor positioning system is considered in [23]. The most efficient solution consists of four RTLS location sensors *LS_A_*, *LS_B_*, *LS_C_*, and *LS_D_* located in workspace corners *A*$\left(x_{A},y_{A},z_{A}\right)$, *B*$\left(x_{B},y_{B},z_{B}\right)$, *C*$\left(x_{C},y_{C},z_{C}\right)$, and *D*$\left(x_{D},y_{D},z_{D}\right)$, (Figure 3 and Table 1).

The top view of the modeled indoor environment, presented in Figure 2, is used to illustrate the azimuth AoA, which is an angular measurement projected on the XY plane. The top view considers a workspace for further analysis, where the height dimension Z is neglected.

The workspace can consist of furnishing and other obstacles. Furthermore, the walls usually are heterogeneous and include windows, doors, *etc.* The materials that are used can also be multifarious. However, all these factors cannot be included in the model in an easy way. Therefore, a heuristic method can help to comprise all these factors into the model and customize it. The fingerprint approach is a possible solution to the problem.

#### 4.2.2. Uncertainty Model

**Figure 4:**
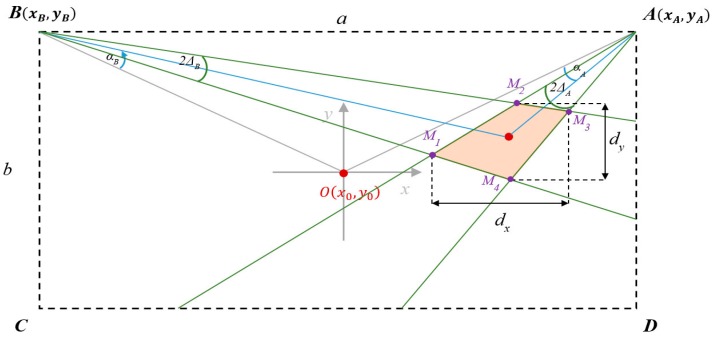
Graphical interpretation of the AoA uncertainty.

The fingerprint map formation, applied for localization using the ranging technique, is based on the model of the indoor environment and on an uncertainty model, both in the same coordinate system. The exemplary analysis concerns a pair of *LS_A_* and *LS_B_* placed in the fixed positions $A\left(x_{A},y_{A}\right)$ and $B\left(x_{B},y_{B}\right)$, and the specified tag T at the position $T\left(x_{i},y_{i}\right)$, shown in Figure 4. The proposed statistical uncertainty model, defines localization uncertainty in a specified position T on the workspace.

We assume that in each tag’s position on the workspace, there are two corresponding sets of angles represented by matrices $AoA_{A}$ and $AoA_{B}$ of N samples of angles of arrival $\alpha_{Ai}\;$and $\alpha_{Bi}$ measured by LSs placed in positions *A* and *B*, respectively. Based on the ranging technique, those sets define N samples of tag position $T_{i}$, specified by intersections of lines corresponding to paths of arrival. Based on experimental data, we assume that the distribution functions of measurement precision and possible interferences of measurement signals are normal. Then, the probability of the single standard deviation of the mean is 68.3%, the angles $\alpha_{Ai}$ and $\alpha_{Bi}$ are within the ranges $\alpha_{Ai}\in \left(\bar{\alpha_{A}}-\sigma_{A},\;\bar{\alpha_{A}}+\sigma_{A}\right)$ and $\alpha_{Bi}\in \left(\bar{\alpha_{B}}-\sigma_{B},\;\bar{\alpha_{B}}+\sigma_{B}\right)$, which define the angles of precision (AoP). Mean values $\bar{\alpha_{A}}$ and$\;\bar{\alpha_{B}}$ represent AoA mean values, which are used to calculate the best estimate of the tag’s position $\hat{\bar{T}}$. Standard deviations of the AoA for both *LS_A_* and *LS_B_* are represented by $\pm \sigma_{A}$ and $\pm \sigma_{B}$, respectively. Thus, the AoA normal distribution of N samples for location sensor *LS_A_* is defined as $N_{A}\left(\bar{\alpha_{A}},\sigma_{A}\right)$.

The distribution function of the intersection of two AoPs (Figure 4) is the multivariate normal distribution as a linear combination of the two normal distributions. The probability that the true value is placed in the common area of these two distributions equals 46.6% as the product of two probabilities of 68.3% corresponding to single standard deviation probability.

The common area of AoPs forms the tetragon of vertices $M_{1-4}$. The area of the tetragon depends on the uncertainty of AoA measurements represented by variances $\sigma_{A}$ and$\;\sigma_{B}$, and on distances $r_{A}$ and $r_{B}$ from LSs to the estimated position $\hat{\bar{T}}$. Then the uncertainty of AoA ranging technique can be estimated in terms of:

- Surface area of the tetragon, which can be calculated using the Shoelace formula [24],
- Lengths $d_{x}$ and $d_{y}$ (Figure 4) corresponding to maximum uncertainty of x and y components, respectively.

The surface area of the tetragon as an estimated uncertainty is the angular measure of precision analogically to Geometric Dilution of Precision (GDoP), which is a measure of positional measurement precision used in satellite navigation.

The uncertainty of the AoA ranging technique can be described by the angular precision non-exclusively assuming the same distribution in the whole workspace. Due to the physical properties of the LS, the best measurements of the AoA are for small angles, which means for the tag placed centrally or near to the calibration point. Then, it is reasonable to combine two components of uncertainty, one constant and one varying. The constant component corresponds to a basic standard deviation $\pm \sigma_{A}$ and $\pm \sigma_{B}$ for *LS_A_* and *LS_B_*, respectively, and can be derived empirically. The varying component increases linearly with coefficient t depending on the angle of inclination |α| to the calibration axis. Absolute angular uncertainty $\Delta_{A}\;$of measure the AoA for the location sensor *LS_A_* is described by equation:

(4) $$\Delta_{A}=\;\sigma_{A}+t\times \left|\bar{\alpha_{A}}\right|$$

where $\sigma_{A}$ is a standard deviation of the AoA measurement, $\bar{\alpha_{A}}$ is a mean angle value of the AoA for the location sensor *LS_A_* and t is a coefficient of the uncertainty varying component. Clearly, if the coefficient t equals zero, then the case of constant uncertainty component over the whole workspace takes place. 

In the further analysis, the localization uncertainty is considered as the surface area of the tetragon defined by vertices *M*_1_–*M*_4_, (Figure 4).

#### 4.2.3. Uncertainty Map

An uncertainty map may be represented by the fingerprint map within the workspace. For this purpose, for the entire workspace, uncertainties are calculated using the AoA statistical geometrical model presented in the previous section.

The workspace of size a×b was sampled with a constant step s in both X and Y directions which resulted in S samples. As a result, a grid of sampling points of the size I×J is formed. Meanwhile, *M* location sensors can create $K=\left(\begin{array}{c} M\\ 2 \end{array}\right)$ different pairs. For each of the *M* pairs of LSs, at each of the S sample positions, the localization uncertainty is calculated. Thus, if for *K* pairs of LSs, the uncertainty maps take a form of multidimensional matrices D of size *I* × *J* × *K*, where the third dimension corresponds to a number of possible pairs. Then the AoA fingerprint map U is calculated using the following formula:

(5) $$\underset{\begin{array}{c} i,j\\ 1\leq i\leq I,1\leq j\leq J\; \end{array}}{\wedge }U\left(i,j\right)=p\in \left\{1,\ldots ,\;K\right\}\;\text{where}\underset{\begin{array}{c} i,j,k\\ 1\leq i\leq I,1\leq j\leq J,\;1\leq k\leq K\; \end{array}}{\wedge }D\left(i,j,U\left(i,j\right)\right)=\;\underset{k\in \left\{1,\ldots ,\;K\right\}}{\min}D\left(i,j,k\right)\;$$

where *U*(*i*,*j*) and *D*(*i*,*j*,*k*) are elements of matrices **U** and **D**, respectively.

For each grid coordinate (*i,j*) from all *K* pairs of LSs, the minimum value of the AoA uncertainty minD(i,j,k) is determined. The fingerprint map, represented by the matrix U shows the distribution of these pairs of LSs for which the AoA uncertainty is minimal.

The fingerprint map U represents a grid of points of given height and known *X, Y* coordinates. If to each of the *K* possible pairs of the LSs we assign a suitable *k-*marker, then the markers can establish the fingerprint map. Neighboring markers of the same type form homogenous groups of markers, which define zones with preferable pairs of LSs.

### 4.3. Online Synthesis

In the online synthesis, actual AoA measurements are compared with the fingerprint map established in the offline phase. The synthesis consists of two stages: the online ranging technique and an estimation algorithm, which determines the final tag’s position. The online synthesis is performed on the workspace, which corresponds to the defined environmental model.

**Figure 5:**
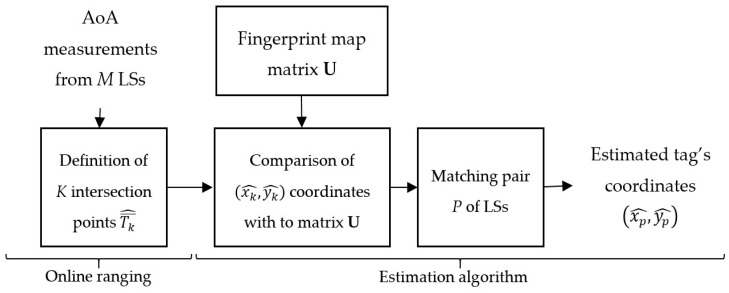
Online synthesis block diagram.

The RTLS sampling frequency determines the measurement cycle in which each of the *M* LSs sequentially measures the corresponding AoA from the tag. For each measurement cycle, the online ranging and estimation algorithms are performed. The block diagram of the online synthesis presented in Figure 5 corresponds to one cycle of online ranging and estimation algorithms.

The online ranging algorithm is based on actual AoA measurements and an environmental model defined during the offline phase. For tag *T*, the ranging algorithm calculates the tag’s position $\hat{\bar{T_{k}}}\left(\hat{x_{k}},\hat{y_{k}}\right)\;$for each possible pair of LSs from *K* available pairs.

Then, the estimation algorithm sets together the online ranging results and the fingerprint map prepared beforehand. The fingerprint map represented by the matrix **U** defines the zones by indicating the preferable pairs of LSs. The set of intersection points $\hat{\bar{T_{k}}}\left(\hat{x_{k}},\hat{y_{k}}\right)$ determined for each of the *K* available LS pairs is compared with the fingerprint map **U**. Thus, each $\hat{\bar{T_{k}}}\left(\hat{x_{k}},\hat{y_{k}}\right)$ point is assigned to the corresponding zone of the preferable pair *p* of LSs. The zone, which includes most of the points is classified as the most precise zone and the zone’s corresponding pair *p* of LSs is selected. The intersection point $\hat{\bar{T_{p}}}$ with coordinates $\left(\hat{x_{p}},\hat{y_{p}}\right)$, derived from the measurements of the *p*-th pair of LSs, becomes the result of the estimation algorithm.

## 5. Implementation and Evaluation

The implementation of the proposed 2D model is done in Matlab 2014b with Signal Processing and Optimization toolboxes.

The implemented solution is evaluated using three different test scenarios for three test environments given in Table 2. The space is sampled with a constant step 25 cm in the X and Y directions resulting in a grid of samples. Since the samples, which are localized beyond the workspace, are filtered out from the analysis, then the sampling coordinates located on the border of the workspace are not in the sampling grid.

In each test scenario, the RTLS consists of four LSs located in the workspace corners and the calibration point located in the center of the workspace. The calibration point constitutes an origin of the coordinate system. For each sampling point, the uncertainty tetragon area is calculated according to the AoA’s estimated angle of precision. The AoA standard deviation value for the specified workspace was statistically determined from results of tests performed in several random locations of workspace for all LSs. The empirically estimated value of the average AoA standard deviation was 0.3°. The implemented model was also tested on four coefficients t∈{0.1, 0.5, 1.0, 2.0} of the uncertainty varying component.

The proposed model is used to estimate the uncertainty for each pair of LSs. Because of test environment symmetry, the uncertainty distribution analysis can be reduced to three of six possible pairs: *AB*, *BD*, *AD.*

To evaluate the method, we compared uncertainty distributions and relevant AoA fingerprint maps for six LS pairs: *AB*, *AC*, *BC*, *AD*, *BD*, *CD*. The evaluation of uncertainty distributions and AoA fingerprint maps was performed for the constant uncertainty component. The influence of the angular uncertainty varying component was evaluated and is presented in the end of this section.

### 5.1. Uncertainty Distribution Evaluation

**Figure 6:**
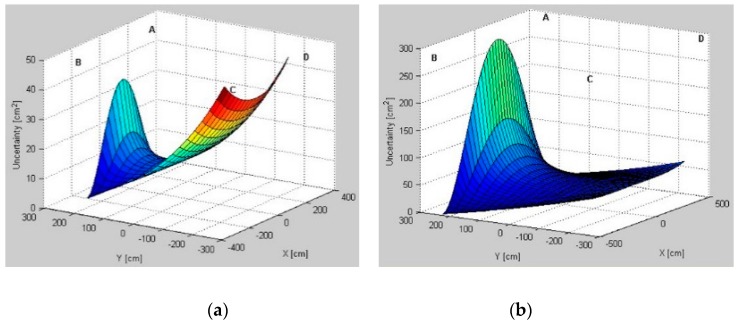

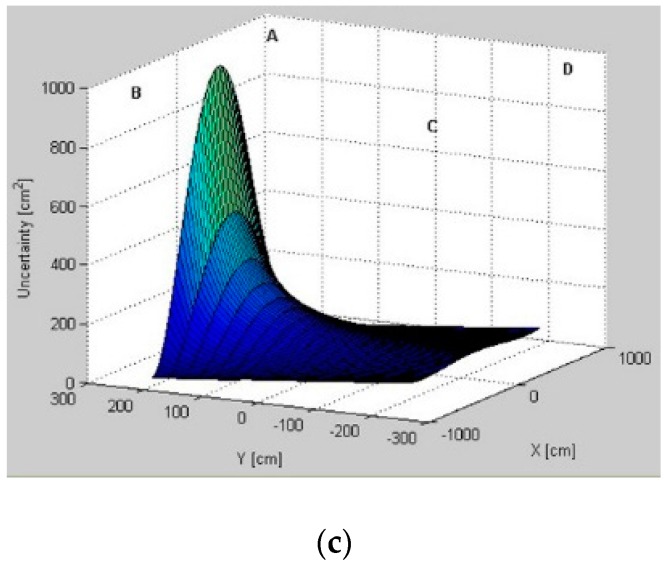
Uncertainty distributions for location sensors *AB* in (**a**) test environment *TE1*; (**b**) test environment *TE2*; and (**c**) test environment *TE3*.

**Figure 7:**
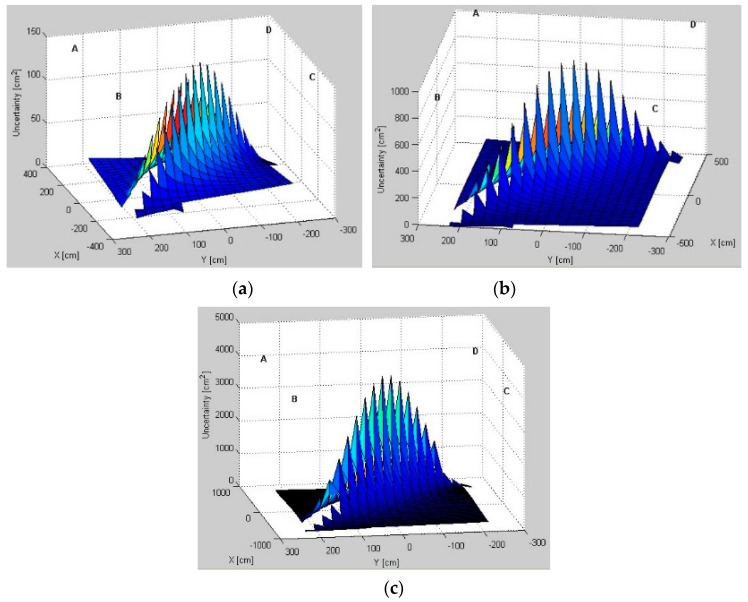
Uncertainty distributions for location sensors *BD* in (**a**) test environment *TE1*; (**b**) test environment *TE2*; and (**c**) test environment *TE3*.

**Figure 8:**
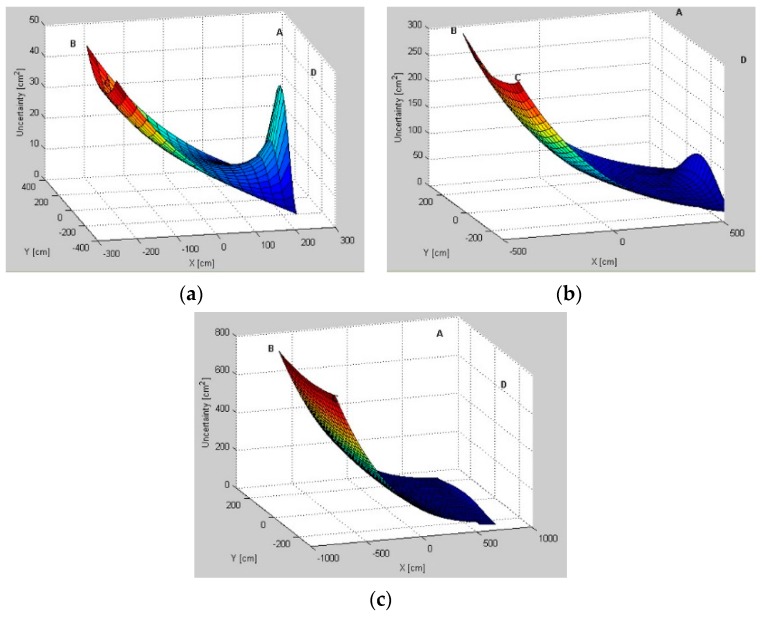
Uncertainty distributions for location sensors *AD* in (**a**) test environment *TE1*; (**b**) test environment *TE2*; and (**c**) test environment *TE3*.

Modeled AoA uncertainty distributions for three pairs, *AB*, *BD*, and *AD*, for different test environments are presented in Figure 6, Figure 7 and Figure 8, respectively. From a comparison of uncertainty maps of the same pair for different test environments, it can be seen that the size of the indoor space significantly influences uncertainty distribution. For the *AB* pair, for the test environment *TE1* the maximum uncertainty is about $45\;{\text{cm}}^{2}$, for the test environment *TE2* it is $280\;{\text{cm}}^{2}$, and finally, the test environment *TE3* has the maximum uncertainty of $750\;{\text{cm}}^{2}$.

For non-square test environments, the level of uncertainty is also affected by pair selection. For the *AB* pair located on a longer room wall, the uncertainty near the LS location wall is very high. For the *AD* pair located on a shorter room wall, the uncertainty increases along with the distance from LSs and, therefore, on the opposite side of the workspace the uncertainty is the highest. Moreover, for all environments, if the active LSs are arranged on diagonal corners, *i.e.*, *BD*, the uncertainty in the central part of the workspace and around the diagonal is very high.

The lowest uncertainty for pairs AB and AD is observable near the active pairs of LSs. Moreover, in the center of the workspace the level of uncertainty is also relatively low. For the diagonal BD pair, the lowest uncertainty is visible near to the non-active *A* and *C* LSs.

### 5.2. Fingerprint Map Evaluation

**Figure 9:**
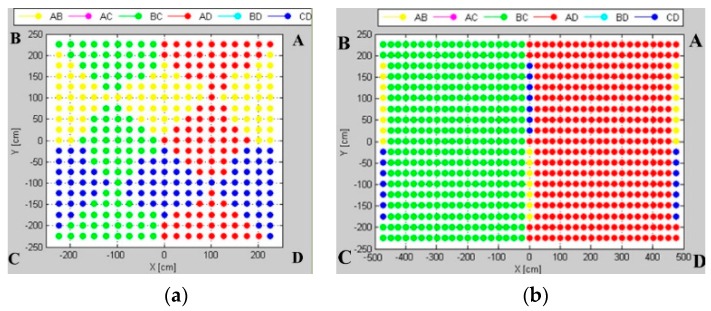

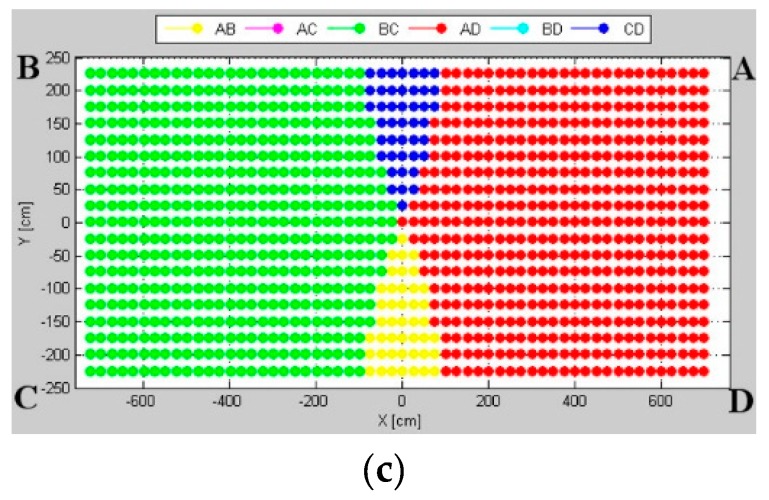
Uncertainty maps for (**a**) test environment TE1; (**b**) test environment TE2; and (**c**) test environment TE3.

The fingerprint map represents a grid of markers, corresponding to the preferable pairs of location sensors for which the AoA uncertainty is smallest at the grid point; the map is presented in Figure 9.

The size of workspace influences the uncertainty map layout. In the test environment *TE1*, the pattern is a symmetric composition of 12 rhombic zones for four pairs arranged on the sides of the workspace, *i.e.*, *AB*, *BC*, *AD*, *CD*. In the test environments *TE2* and *TE3*, the patterns are generally divided into two zones: green (LSs *BC*) and red (LSs *AD*), *i.e.*, the pairs of sensors located on the shorter dimension. Along with the increase in the aspect ratio of the rectangular room, pairs *AB* and *CD* become more important in the central zone of the pattern. The level of uncertainty for the pairs arranged in diametrical corners, *i.e.*, *AC* and *BD*, is relatively high, thus, they do not contribute to the uncertainty map. 

### 5.3. Evaluation of Varying Components of Angular Uncertainty

An effect of the angular uncertainty varying component is investigated in a scenario similar to the real experiment scenario described in Section 6.1, and it means that the *AB* pair is examined on the test environment *TE2* with the reference point slightly moved from the workspace center. The varying component emphasizes a variation of uncertainty distribution caused by the sensor’s varying sensitivity on different AoAs. Thus, the uncertainty of the AoA measurement of a tag located near the calibration point is smaller than for a tag located on the workspace borders.

**Figure 10:**
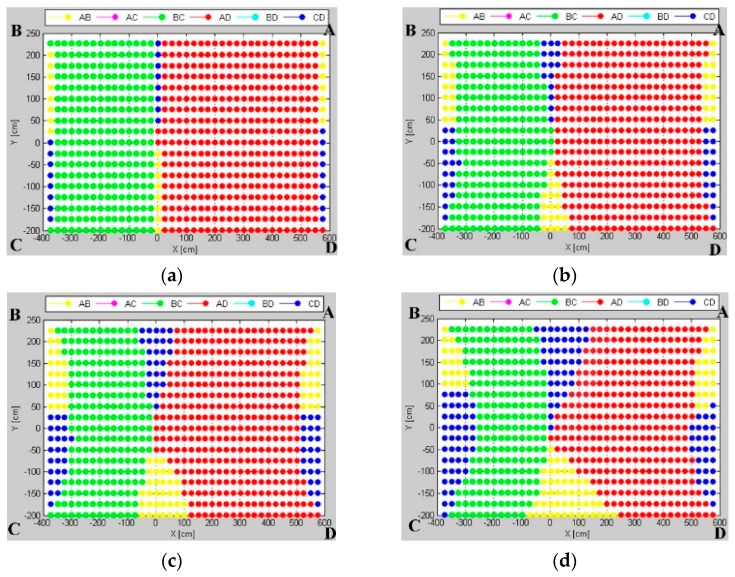
Uncertainty map for test environment TE2 with different uncertainty varying component coefficients: (**a**) 0.1; (**b**) 0.5; (**c**) 1.0; and (**d**) 2.0. The reference point was not located symmetrically.

The investigated influence of the angular uncertainty-varying component on the uncertainty maps is presented in Figure 10. For a small value of coefficient *t* from Equation (4), the pattern mainly consists of markers corresponding to *AD* and *BC* pairs arranged on the shorter sides of the workspace. Along with an increasing value of the coefficient *t* of the varying component, *AB* and *CD* pairs, located on the longer sides, increase their contribution to the map, especially around the calibration point and along the shorter sides.

Simulation results show that the calibration point becomes a demarcation point around which the pattern zones are specified. Along with an increasing value of coefficient *t* of the varying component, the pattern zones evolve, but the calibration point still defines the demarcation point. 

## 6. Verification

The proposed method was verified by physical experiments, evaluating uncertainty distributions for different pairs. Moreover, the experimental fingerprint maps illustrating the workspace zones with preferable pairs were analyzed.

To be able to verify the proposed customized technique, definition of the coherent uncertainty measures, both for the simulation and for the physical experiment, is needed. In simulations, the proposed model of uncertainty is defined as the tetragon area presented in Figure 4, which depends on the AoA and distances from the location sensors. In the experimental approach, it was assumed that X and Y components of the standard error define the uncertainty of the tag’s position estimate. For this application, the 2D standard error takes the form of an ellipse area, the axes of which represent X and Y components of standard deviations.

The physical experiment was carried out on a Ubisense Real Time Localization System Series 7000, consisting of four LSs connected to a PC, and tags, which communicate with LSs on the telemetry channel 2.4 GHz and transmit localization pulses on UWB channel 6–8 GHz. The used tags were Ubisense Compact Tags with the maximum tag update rate 33.75 Hz. LSs were connected with Ubisense Location Platform 2.1 software on a PC by Ethernet. The RTLS operates in AoA, Time Difference of Arrival (TDoA), and RSS physical measurements, however, just AoA raw measurements without filtration are analyzed.

### 6.1. Physical Experiment Arrangement

**Figure 11:**
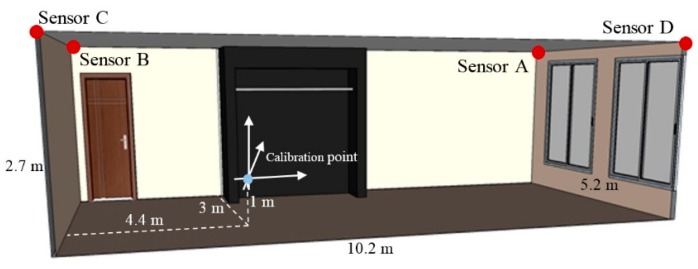
Workspace model where the physical experiment was carried out.

**Figure 12:**
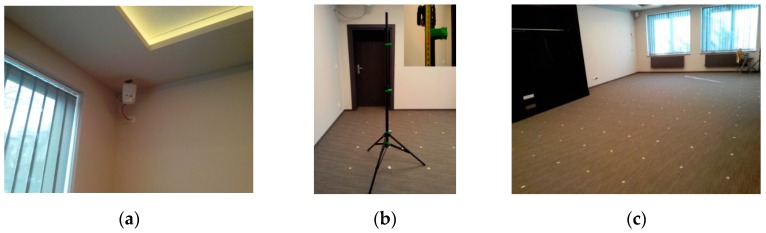
Photos of (**a**) RTLS sensor; (**b**) tripod with mounted tag; and (**c**) indoor environment.

The physical experiment was performed in the workspace of the size 10.2 m×5.2 m×2.7 m, as presented in Figure 11. In the workspace, there were two large windows located on the shorter wall, one entrance door, and a wardrobe placed at the middle of the longer wall. The calibration point was located at a position 4.4 m×3 m×1 m from the corner on the floor near the entrance door. The LS, presented in Figure 12a, was installed in the corner on the ceiling. The tag was mounted on a 1 m high tripod (Figure 12b). 

The whole room was sampled with a 0.5 m step in all directions, which resulted in the sampling grid of 168 samples. The workspace with marked sampling positions is presented in Figure 12c. The area where the wardrobe was mounted was excluded from the workspace. In each position, 100 samples of the AoA from all LSs were collected.

The simulation test environment *TE2* corresponds to the workspace size of the physical experiment.

### 6.2. Standard Error Distribution

**Figure 13:**
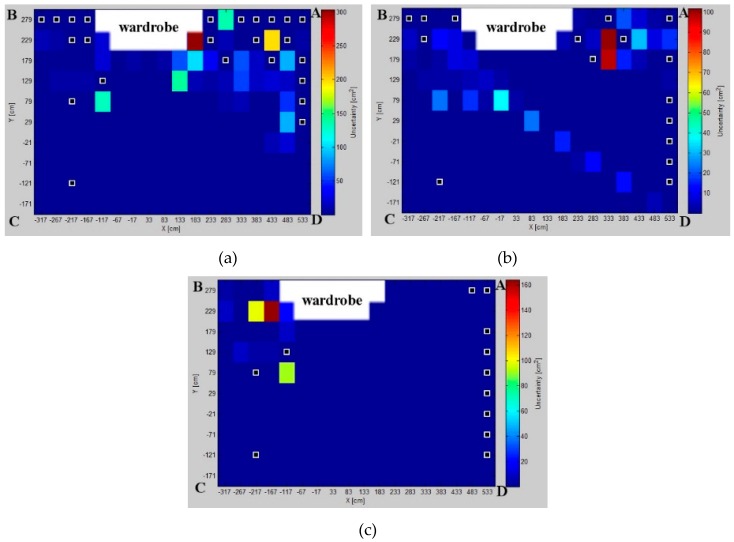
Standard error distributions for pairs: (**a**) *AB*; (**b**) *BD*; and (**c**) *AD*. Sampled positions with white frame markers show the sampling points with gross error.

For each pair, in each sampled position, the standard error was calculated, which resulted in a map of standard error distributions, see Figure 13 for three LS pairs. Because of the obstacles and the environmental interferences, some gross errors and samples out of the workspace were filtered out. The impact of the windows and wardrobe is noticeable in the results. For instance, the zone in the upper left-hand corner is visibly overshadowed by the wardrobe. 

For most of the sampled positions, the localization standard error was less than $10\;{\text{cm}}^{2}$, which denotes high precision. Considering experimental results, the best arrangement of the LSs is of the *AD* pair, showing not only the best precision, but also for this pair, the number of points, characterized by the gross error, was the lowest. In the case of the LSs arranged on diametrical corners, the results are consistent with simulated results. For the *BD* pair, the localization standard error is biggest on the diagonal, which is also in line with an expectation from simulation.

### 6.3. Fingerprint Map

**Figure 14:**
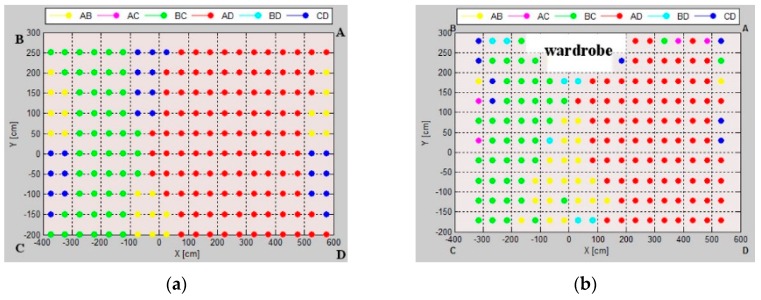
Uncertainty map from: (**a**) simulation; (**b**) physical experiment.

The fingerprint map shown in Figure 14, represents a grid of markers of preferable pairs of LSs for which the experimental localization standard error is lowest. On this map, there may be specified three main zones for three pairs: *BC* and *AD* arranged on shorter sides and the *AB* pair on the longer side of the indoor space. The experimental results are in line with simulated results; this indicates the calibration point as a demarcation point around which the pattern zones are specified. In Figure 10, to the right of the calibration point, minimum localization standard error is noticed mostly for the *AD* pair. To the left of the calibration point, the *BC* pair shows the most precise AoA localization. A relatively small zone located centrally is visible, where the minimal standard deviation for the *AB* pair is arranged on the longer wall of the room, as it is in the simulated map. However, due to obstacles, the contribution of the *CD* pair is not as noticeable as it is in simulation.

The likeness of uncertainty maps derived from simulation and physical experiment proves that the analytical method based on the AoA statistical geometrical model is a good representation of AoA uncertainty distribution in an indoor space.

## 7. Validation

To validate the proposed solution, the AoA statistical geometrical model of uncertainty of the constant component was compared with real measurements. To quantitatively measure the correlation between the proposed analytical uncertainty model and the experimental results of the localization standard error, at each sample position in the workspace, a matching ratio was determined as a percentage of experimental locations inside the theoretical tetragon estimated from the proposed model.

**Figure 15:**
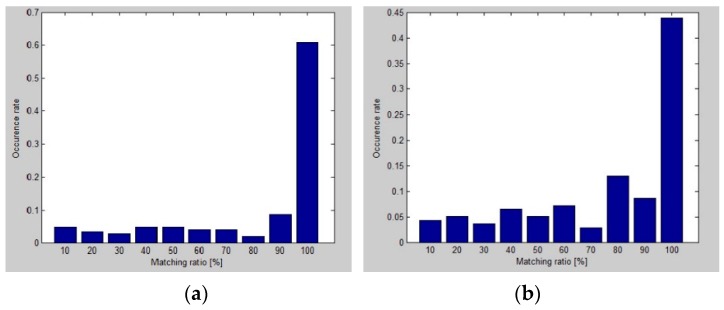

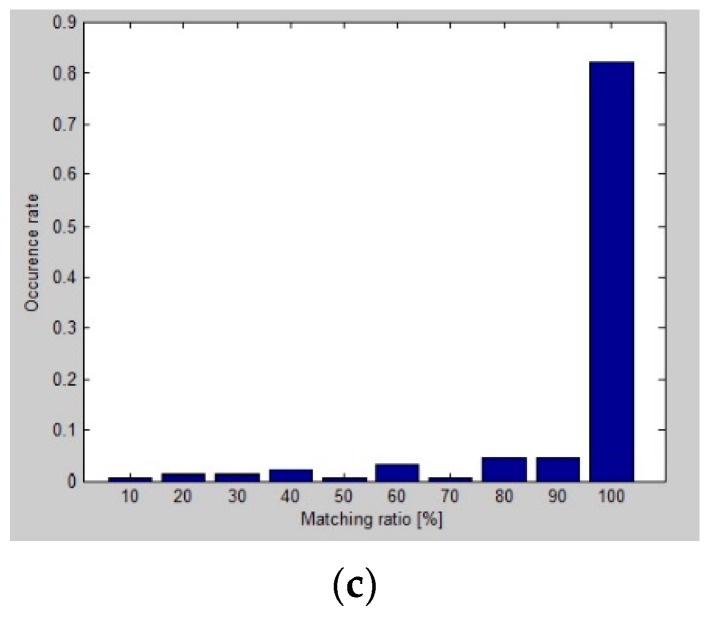
Histograms with matching ratio results for (**a**) location sensors *AB*; (**b**) location sensors *BD*; and (**c**) location sensors *AD*.

Histograms with the matching ratio *vs.* the occurrence rate for three analyzed LS pairs are presented in Figure 15. It shows that for all considered pairs, and the vast majority of sample positions, the matching ratio is very high. For the *AD* pair located on the short wall, about 80% of the sampled positions meet the matching ratio, close to 100%. Even for the worst *BD* pair, the 100% matching ratio level is achieved at about 45% of the sampled positions on the workspace.

**Figure 16:**
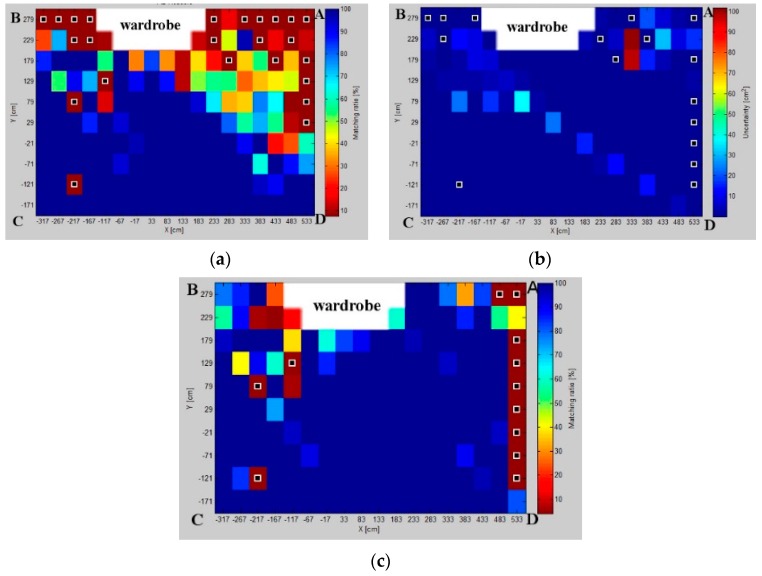
Validation of proposed model with physical experiment results for pairs: (**a**) *AB*; (**b**) *BD*; and (**c**) *AD*. Sampled positions with white frame markers show the sampling points with gross error.

The matching ratio maps for three considered pairs, presented in Figure 16, show the matching ratio distribution on the workspace. This map depicts zones with the best matching ratio for different pairs, but can be also used to detect the zones with the lowest matching ratio caused by higher interferences.

The matching ratio maps reveal the environmental interferences caused by obstacles or constructions, such as doors, furniture, and windows. In Figure 16, the wardrobe shades the upper right and left zones, of the workspace. Furthermore, the interferences from the window and doors can be also noticed. However, even in cases of strong environmental interferences visible in Figure 16, the matching ratios are statistically valid. Figure 15b proves that the physical results are consistent with the theoretical model.

## 8. Results Discussion

The simulation results show how the uncertainty depends on the arrangement of LSs and on the size and shape of the workspace (Figure 6, Figure 7 and Figure 8). The uncertainty distribution maps are specific for each LS pair, and it is proved that the level of uncertainty increases along with an increasing aspect ratio of the rectangular workspace. The diagonal pairs are the most adverse arrangement of LSs, showing the peak of the uncertainty around the workspace diagonals. Based on the simulation results, it can be seen that the uncertainty increases along with the distance from active LSs.

The fingerprint maps presented in Figure 10 illustrate the effect of the workspace size in terms of the aspect ratio of a rectangular workspace. One can observe how a number and size of pattern zones evolve. For the workspace with a small aspect ratio, the most suitable *BC* and *AD* pairs are located on the shorter walls of the room. Along with increasing the workspace’s aspect ratio, the *AB* and *CD* pairs, arranged on the longer dimension, contribute more, especially in the central zone of the pattern. 

The validity of the proposed method is proven by a comparison of the simulated and experimental results presented in Figure 13. The results of both approaches confirm that the best localization precision is achieved for the *AD* pair. Likewise, the experimental results justify the respective simulation results for the diagonal pairs, where the uncertainty is the worst. 

The impact of the indoor obstacles, such as windows, doors, wardrobe *etc.* is noticeable from experimental results. The zone along the walls with the wardrobe and windows required preprocessing, due to the strong interferences from the obstacles.

The fingerprint map of real measurements, shown in Figure 14, consists of three main zones for three pairs: *BC*, *AD*, *AB*. It is noticeable that the calibration point is a kind of demarcation around which the pattern zones are distributed. Generally, the pairs *BC* and *AD*, along the shorter walls, contribute to the zones with the high AoA precision on their sides of the calibration point. In the central zone of pattern, the best results are acquired for the *AB* pair.

On the entire workspace, the fingerprint maps from the physical experiment are correlated with simulated ones, and Figure 16 proves that at most of the grid points, the matching between both approaches is at an expected level and statistically valid. Even for the worst *BD* pair, the maximal 100% matching ratio is achieved at about 45% of the sample positions on the workspace.

## 9. Conclusions

The performance enhancement of the indoor AoA-based RTLS, by applying an analytical model of the AoA uncertainty, was accomplished by customizing the RTLS using the fingerprinting technique along with the AoA ranging technique. To execute the research objectives, the model of the fingerprinting technique applied to indoor environment scenarios was implemented in Matlab. The proposed analytical uncertainty model was evaluated and verified by a set of simulations and physical experiments.

The results derived from the simulations and physical experiments validate the analytical fingerprint maps as an adequate performance of RTLS in terms of precision. The proposed analytical uncertainty model is a suitable way to customize RTLS.

The results of simulations in line with the experiments show how the localization precision of the AoA-based RTLS depends on the LSs’ arrangement along with the workspace size and shape. For the analyzed test scenarios, the simulation and physical experiment indicate that the pairs located on the shorter sides of the workspace are the most reliable and suitable for precise AoA localization. However, around the calibration point, even the pairs located along the long sides contribute to the fingerprint map, especially for the space with the high aspect ratio of rectangular spaces. The uncertainty level for the diagonal pairs is relatively high, particularly along the diagonals. 

The experimental results evidence the significant interferences from the indoor obstacles, such as furniture, doors, and windows. However, the proposed model complies with the references with statistically valid robustness.

Moreover, the presented studies show how the size and shape of the workspace affect both a number and shapes of pattern zones of the fingerprint map.

The study depicts an important role of the calibration point as a demarcation point around which pattern zones are placed. It justifies why the calibration point should be located as centrally as possible.

To complement this research, which focuses mostly on localization precision, further research may also concern the accuracy of AoA-based RTLSs. The accuracy, interpreted as a systematic error, can be compensated based on the previously prepared accuracy map. Such research, combined with the results of this paper, may provide a holistic approach to the AoA-based RTLS.

Improving the classification method determining optimal pairs of LSs, should enhance the estimation algorithm reliability. Such algorithm may be implemented and tested on various indoor environments to prove its versatility.

It seems that modeling of the localization uncertainty map is also possible for the signal of ToA or TDoA, which can be used along with the ranging technique. Therefore, the proposed analytical fingerprint method may be implemented in the TDoA-based RTLS. A similar localization uncertainty map, but based on a heuristic approach, can even be established for RSSI signals and, accordingly, a heuristic fingerprint solution can be used in RSSI-based RTLS.

## Figures and Tables

**Table 1:** Location sensors arrangement.

LS	General Coordinates	Specific Coordinates
*LS_A_*	$A\left(x_{A},y_{A},z_{A}\right),$	A(a/2;b/2;c/2)
*LS_B_*	$B\left(x_{B},y_{B},z_{B}\right)$	B(−a/2; b/2; c/2)
*LS_C_*	$C\left(x_{C},y_{C},z_{C}\right)$	C(−a/2;−b/2; c/2)
*LS_D_*	$D\left(x_{D},y_{D},z_{D}\right)$	D(a/2; b/2; c/2)

**Table 2:** Environmental scenarios.

Environmental Scenario	Size *a* × *b* [cm × cm]
Test environment *TE1*	500 × 500
Test environment *TE2*	500 × 1000
Test environment *TE3*	500 × 1500

## Abbreviations

The following abbreviations are used in this manuscript:

AoA	Angle of Arrival
AoP	Angle of Precision
DoP	Dilution of Precision
GDoP	Geometric Dilution of Precision
ILS	Indoor Localization System
LoS	Line-of-sight
LS	Location Sensor
NLoS	Non-line-of-sight
RSS	Received Signal Strength
RTLS	Real-time Locating Systems
TDoA	Time Difference of Arrival
ToA	Time of Arrival
UWB	Ultra Wideband
WSN	Wireless Sensor Network

## References

[1] ISO/IEC 24730-1:2014—Information Technology—Real-Time Locating Systems (RTLS) 2014. http://www.iso.org/iso/catalogue_detail.htm?csnumber=59801.

[2] Kamel Boulos M.N., Berry G. (2012). Real-time locating systems (RTLS) in healthcare: A condensed primer. Int. J. Health Geogr..

[3] Fisher J.A., Monahan T. (2012). Evaluation of real-time location systems in their hospital contexts. Int. J. Med. Inf..

[4] Bowen M.E., Craighead J., Wingrave C.A., Kearns W.D. (2010). Real-Time Locating Systems (RTLS) to improve fall detection. Gerontechnology.

[5] Ma X., Liu T. The application of Wi-Fi RTLS in automatic warehouse management system. Proceedings of the 2011 IEEE International Conference on Automation and Logistics (ICAL).

[6] Cho H., Kim T., Park Y., Baek Y. Enhanced Trajectory Estimation Method for RTLS in Port Logistics Environment. Proceedings of the 2012 IEEE 14th International Conference on High Performance Computing and Communication and 2012 IEEE 9th International Conference on Embedded Software and Systems (HPCC-ICESS).

[7] Deak G., Curran K., Condell J. (2012). A survey of active and passive indoor localisation systems. Comput. Commun..

[8] Malik A. (2009). RTLS for Dummies.

[9] Kim D.K., Ha J.H., Kim P.J., You K.H. TDOA/AOA localization in RFID system using dual indirect Kalman filter. Proceedings of the 2011 IEEE/SICE International Symposium on System Integration (SII).

[10] Tay B., Liu W., Zhang D.H. Indoor Angle of Arrival positioning using biased estimation. Proceedings of the 7th IEEE International Conference on Industrial Informatics (INDIN).

[11] Wen C.-Y., Chan F.-K. (2010). Adaptive AOA-Aided TOA Self-Positioning for Mobile Wireless Sensor Networks. Sensors.

[12] Park J.W., Park D.H., Lee C. (2011). Angle and ranging based localization method for ad hoc network. J. Supercomput..

[13] Wang Y., Ho K.C. (2015). An Asymptotically Efficient Estimator in Closed-Form for 3-D AOA Localization Using a Sensor Network. IEEE Trans. Wirel. Commun..

[14] Dempster A.G. (2006). Dilution of precision in angle-of-arrival positioning systems. Electron. Lett..

[15] Myong S. (2011). Location Error Analysis of an Active RFID-Based RTLS in Multipath and AWGN Environments. ETRI J..

[16] Crespo G., Teniente J., Ederra I., Gonzalo R. Experimental study of the antenna influence in RTLS based-on RFID. Proceedings of the 2012 6th European Conference on Antennas and Propagation (EUCAP).

[17] Ma Z., Ho K.C. (2014). A Study on the Effects of Sensor Position Error and the Placement of Calibration Emitter for Source Localization. IEEE Trans. Wirel. Commun..

[18] Edelhauser T., Janiak M., Kókai G. Environment-based measurement planning for autonomous RTLS configuration. Proceedings of the IEEE 2010 NASA/ESA Conference on Adaptive Hardware and Systems (AHS).

[19] Wu Z.-H., Han Y., Chen Y., Liu K.J.R. (2015). A Time-Reversal Paradigm for Indoor Positioning System. IEEE Trans. Veh. Technol..

[20] Lin P., Li Q., Fan Q., Gao X., Hu S. (2014). A Real-Time Location-Based Services System Using WiFi Fingerprinting Algorithm for Safety Risk Assessment of Workers in Tunnels. Math. Probl. Eng..

[21] Jachimczyk B., Dziak D., Kulesza W. (2013). Performance analysis of an RFID-based 3D indoor positioning system combining scene analysis and neural network. Sci. Pap. Fac. Electr. Control Eng. Gdansk Univ. Technol..

[22] Outemzabet S., Nerguizian C. Accuracy enhancement of an indoor ANN-based fingerprinting location system using Kalman filtering. Proceedings of the 2008 IEEE 19th International Symposium on Personal, Indoor and Mobile Radio Communications.

[23] Jachimczyk B., Dziak D., Kulesza W.J. RFID—Hybrid Scene Analysis-Neural Network system for 3D Indoor Positioning optimal system arrangement approach. Proceedings of the 2014 IEEE International Instrumentation and Measurement Technology Conference (I2MTC).

[24] Shoelace Formula. https://en.wikipedia.org/wiki/Shoelace_formula.

